# Estimating infant age from skull X-ray images using deep learning

**DOI:** 10.1038/s41598-024-64489-4

**Published:** 2024-07-18

**Authors:** Heui Seung Lee, Jaewoong Kang, So Eui Kim, Ji Hee Kim, Bum-Joo Cho

**Affiliations:** 1 Department of Neurosurgery, College of Medicine, Hallym University Sacred Heart Hospital, Hallym University, 22, Gwanpyeong-Ro 170Beon-Gil, Dongan-Gu, Anyang-Si, Gyeonggi-Do 14068 Republic of Korea; 2 Interdisciplinary Program for Bioinformatics, Graduate School, Seoul National University, Seoul, Republic of Korea; 3 Medical Artificial Intelligence Center, Hallym University Medical Center, Anyang, Republic of Korea; 4 Department of Ophthalmology, College of Medicine, Hallym University Sacred Heart Hospital, Hallym University, 22, Gwanpyeong-Ro 170Beon-Gil, Dongan-Gu, Anyang-Si, Gyeonggi-Do 14068 Republic of Korea

**Keywords:** Infantile skull, Skull suture, Infant age, Craniosynostosis, X-ray, Medical research, Paediatric research, Computational biology and bioinformatics, Image processing, Medical imaging, Bone imaging, Radiography

## Abstract

This study constructed deep learning models using plain skull radiograph images to predict the accurate postnatal age of infants under 12 months. Utilizing the results of the trained deep learning models, it aimed to evaluate the feasibility of employing major changes visible in skull X-ray images for assessing postnatal cranial development through gradient-weighted class activation mapping. We developed DenseNet-121 and EfficientNet-v2-M convolutional neural network models to analyze 4933 skull X-ray images collected from 1343 infants. Notably, allowing for a ± 1 month error margin, DenseNet-121 reached a maximum corrected accuracy of 79.4% for anteroposterior (AP) views (average: 78.0 ± 1.5%) and 84.2% for lateral views (average: 81.1 ± 2.9%). EfficientNet-v2-M reached a maximum corrected accuracy 79.1% for AP views (average: 77.0 ± 2.3%) and 87.3% for lateral views (average: 85.1 ± 2.5%). Saliency maps identified critical discriminative areas in skull radiographs, including the coronal, sagittal, and metopic sutures in AP skull X-ray images, and the lambdoid suture and cortical bone density in lateral images, marking them as indicators for evaluating cranial development. These findings highlight the precision of deep learning in estimating infant age through non-invasive methods, offering the progress for clinical diagnostics and developmental assessment tools.

## Introduction

As neonates grow, the cranium undergoes significant changes, such as the narrowing of cranial sutures and the closure of fontanels, which are critical markers of normal cranial development^1,2^. Given that craniosynostosis—a condition characterized by the premature fusion of cranial sutures, affecting approximately 1 out of every 2500 live births—can lead to severe neurodevelopmental impairments if left untreated, the need for accurate diagnostic tools for its early detection is crucial^3–6^. Furthermore, in developing diagnostic tools capable of distinguishing such pathological conditions, it is imperative first to have tools that provide information serving as criteria for indicating normal cranial development. However, the lack of comprehensive research on these developmental milestones has led to a scarcity of reference data, making it challenging to assess whether an infant's cranial growth aligns with normal chronological changes.

Plain skull X-ray imaging, with its low radiation exposure and non-invasiveness, offers a valuable resource for evaluating cranial development, yet its potential has been underutilized in the context of age estimation.

With the advent of convolutional neural network (CNN) models, deep learning has revolutionized the ability to classify and interpret medical images with precision surpassing traditional methods^7,8^. This technological advancement opens new avenues for the application of plain skull X-rays in the precise estimation of postnatal age, facilitating the early detection of cranial anomalies such as craniosynostosis.

Therefore, this study aims to assess the utility of a deep learning model developed to predict the postnatal age of infants using plain skull X-ray images, evaluating its applicability in clinical medicine and various related fields. By integrating class activation mapping (CAM) into the deep learning model^9^, this research seeks not only to improve the accuracy of age estimation but also to provide a novel tool that facilitates the assessment of both normal and pathological cranial development. Thus, the model aims to make a significant contribution in areas such as pediatric care, forensic science, and archaeological research.

## Methods

### Data collection

Infants under 12 months of age who underwent plain skull X-rays for head trauma evaluation from January 2010 to December 2021 were included in this study. Patients with congenital cranial malformations were excluded. The study was approved by the Institutional Review Board of Hallym University Sacred Hospital (No. 2023-01-002). Informed consent was waived due to the retrospective nature of the study by the Institutional Review Board of Hallym University Sacred Hospital (No. 2023-01-002), and all image data were anonymized.

All plain skull X-ray images (74 kVp, 200 mA, 100 ms) were taken using a digital radiographic device (GC85A, SAMSUNG, Korea) and retrieved from the Picture Archiving and Communication System (PACS, Infinite version 3.0.9) of the institution in DICOM format and converted into .png format. Personal information and annotations were removed during the conversion process to ensure patient confidentiality, and images that were not properly focused were systematically excluded from the database. To maintain the integrity of our dataset, the acquired images were evaluated by the neurosurgical expert (H.S.L.), who decisively excluded AP, Towne, and lateral view radiographs that deviated significantly from the standards.

Additionally, the study utilized another 864 images of 216 distinct patients from the same institution for external validation. These images were obtained from skull X-ray examinations performed over the period from January 2017 through December 2021. The mean vertical resolution of the internal dataset was 2046 ± 80 (1382–2177) pixels, while the mean horizontal resolution has 1703 ± 74 (1382–2177) pixels. In the external dataset for validation, the mean vertical resolution has 1991 ± 188 (1453–2177) pixels, and the mean horizontal resolution has 1641 ± 181 (1160–1814) pixels.

The skull X-ray images included four different types: anteroposterior (AP), Towne, right lateral, and left lateral views. Some patients had all four types of X-rays, while others had only some of them. Before the study, the X-ray views were categorized into two groups: the AP view dataset, which included both AP and Towne views, and the lateral view dataset, comprising right and left lateral views. Two separate deep learning models were independently developed for these datasets.

### Dataset construction

Each image was labeled according to the patient's age group, categorized into 12 categories by month of age. As presented in Table 1, the entire dataset was divided into three subsets: training, validation, and test datasets, using random sampling at an 8:1:1 ratio. These sub-datasets were mutually exclusive. The validation dataset was used to determine the optimal training process point. Sampling was performed stratified by age groups to maintain consistent data proportions in each subset. To enhance performance reliability, dataset splitting was carried out three times with three different seeds to train deep learning models separately.

**Table 1:** Data composition of enrolled plain skull X-rays of anterior–posterior (AP) and lateral views in the internal datasets.

	Whole subjects			Whole Dataset (numbers)				Training set (numbers)				Validation set (numbers)				Test set (numbers)			
				AP view		Lateral view		AP view		Lateral view		AP view		Lateral view		AP view		Lateral view	
	Patients	Mean age (days)	Male	Patients	Images	Patients	Images	Patients	Images	Patients	Images	Patients	Images	Patients	Images	Patients	Images	Patients	Images
Overall	1343	205 ± 98	711 (53%)	1336	2401	1321	2532	1059	1897	1045	2001	138	253	138	261	139	251	138	270
Postnatal days																			
0–30	66	13 ± 10	43 (65%)	65	78	55	92	51	59	43	75	7	10	6	8	7	9	6	9
31–60	63	44 ± 9	32 (51%)	63	106	62	119	49	84	48	93	7	12	7	12	7	10	7	14
61–90	73	75 ± 8	42 (58%)	73	131	72	138	57	101	56	106	8	15	8	16	8	15	8	16
91–120	74	106 ± 8	40 (54%)	73	129	72	142	57	102	56	110	8	14	8	16	8	13	8	16
121–150	107	136 ± 8	51 (48%)	107	197	106	200	85	160	84	156	11	18	11	22	11	19	11	22
151–180	149	167 ± 9	84 (56%)	148	268	148	288	118	212	118	230	15	27	15	29	15	29	15	29
181–210	142	194 ± 8	68 (48%)	141	256	141	273	111	202	111	216	15	28	15	28	15	26	15	29
211–240	157	224 ± 9	76 (48%)	157	293	156	299	125	230	124	235	16	31	16	32	16	32	16	32
241–270	139	256 ± 9	71 (51%)	139	259	138	268	111	205	110	213	14	27	14	27	14	27	14	28
271–300	123	283 ± 9	67 (54%)	121	225	123	238	96	178	97	187	12	23	13	25	13	24	13	26
301–330	110	313 ± 9	60 (55%)	110	201	110	211	88	159	88	169	11	21	11	20	11	21	11	22
330–365	140	343 ± 8	77 (55%)	139	258	138	264	111	205	110	211	15	27	14	26	14	26	14	27

### Data pre-processing

**Figure 1:**
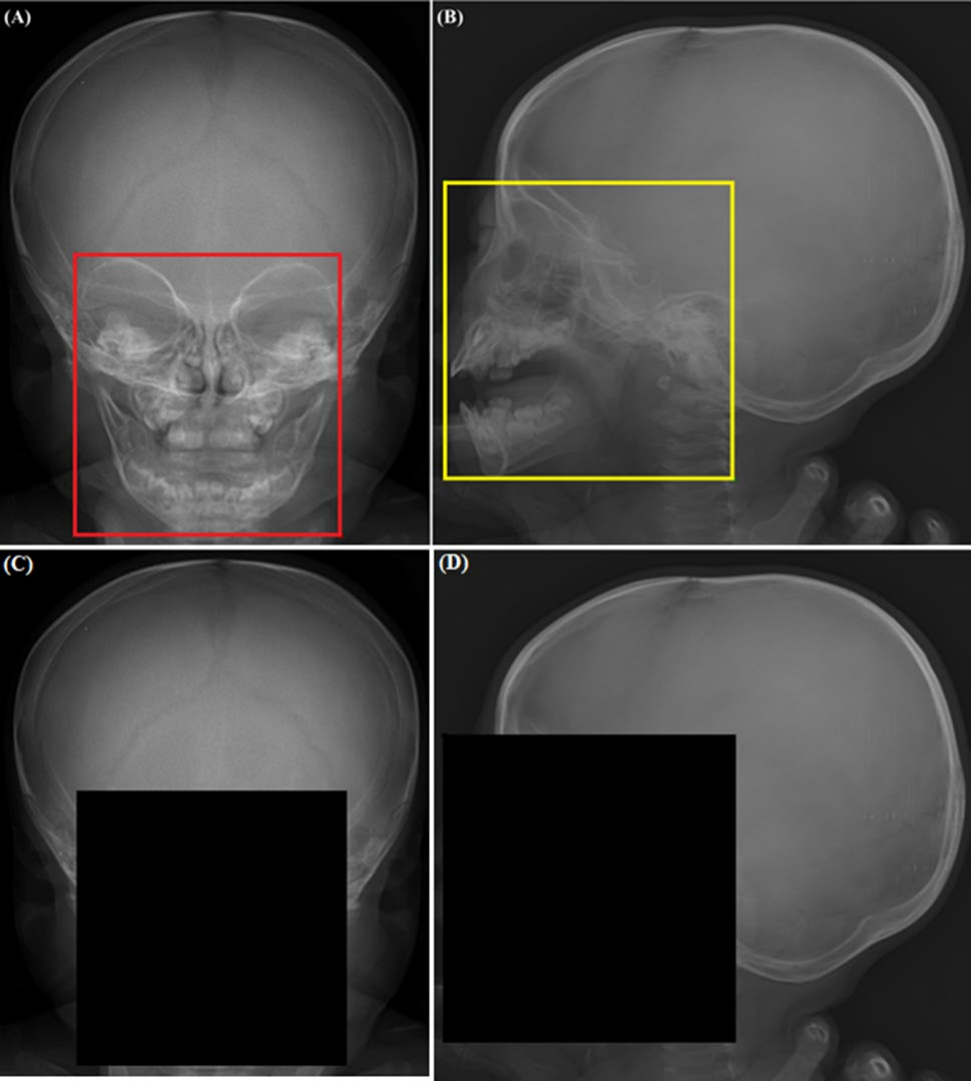
The defined region of exclusion (ROE) in the skull X-ray for image tailoring. (**A**) Anteroposterior (AP) or Town’s view skull X-ray showing the defined ROE. The borders of the ROE extend from the upper margin of the supraorbital rim to the lower margin of the mandible. (**B**) Lateral skull X-ray with the ROE including the supraorbital rim, the foremost part of the mandible, and the posterior margin of the cervical spinous process. (**C**) Post-processed AP or Town’s view skull X-ray. The region below the upper margin of the ROE has been removed. (**D**) Post-processed lateral skull X-ray. A square box, defined by the upper and right margins of the ROE, has been removed.

To eliminate potential age prediction biases unrelated to the skull, all images were pre-processed to hide teeth and paranasal sinus areas. The region of exclusion (ROE) containing the orbital and mandibular regions in the skull X-ray was identified. The border lines of the ROE were defined as follows:

1. on the AP or Town’s skull X-ray image, they encompassed the upper margin of the supraorbital rim and the lower margin of the mandible (Fig. 1A)
2. on the lateral skull X-ray image, they included the supraorbital rim, the foremost part of the mandible, and the posterior margin of the cervical spinous process (Fig. 1B).

The defined area on each of 293 skull X-ray images was labeled as ROE by a neurosurgery expert (H.S.L). The entire ROE dataset was divided into training, validation, and test datasets through random sampling with a ratio of 8:1:1. The MobileNetV3 model was trained for object detection of the labeled ROE. Regarding training parameters, the Adam optimizer was used with an initial learning rate of 1e − 3 and batch size of 16. Subsequently, post-processing was performed on all images to eliminate the detected ROEs based on the following criteria:

1. on AP or Town’s skull X-ray, the region below the upper margin of the ROE was removed (Fig. 1C)
2. on the lateral skull X-ray, the square box defined by the upper margin of the ROE and right margin of the ROE was removed (Fig. 1D).

All tailored images were then reviewed by a neurosurgeon (H.S.L.) and adjusted for any misprocessing. After tailoring the region of interest (ROI) in the images, all images were center-symmetrically zero-padded into square shapes to match the longer side of the width and height. Bi-linear interpolation was applied to the transformed square images of different sizes to resize them to a uniform size of 1024 × 1024 pixels. Min–max normalization was applied to normalize all images.

### Training CNN models

To construct deep-learning models, two different CNN architectures, DenseNet-121 and EfficientNet-V2-M, were adopted. DenseNet-121 has an improved algorithm for feature representation and learning efficiency and has been effective at medical image classification^10^, and EfficientNet-V2-M, which has been relatively recently introduced and has shown higher performance in general image classification tasks with low computational cost^11,12^. In brief, DenseNet consists of dense blocks linking the feature map of previous layers together, while the EfficientNet-V2-M model searches for the most effective CNN architecture using neural architecture search, similar to EfficientNet. DenseNet-121 and EfficientNet-V2-M had previously been trained with the ImageNet dataset and were fine-tuned by unboxing the weights^11–13^. All layers were unfreezed, allowing fine-tuning of every layer in the network.

The batch size was set at 8 for DenseNet-121 and 4 for EfficientNet-V2-M, the maximum capacity that the GPU memory of our hardware could handle with each architecture. Categorical cross-entropy was used as the loss function, and the Adam optimizer was applied^14^. The initial learning rate was set to 0.0001 and was reduced by a factor of 0.1 every 10 epochs. Early stopping was employed after the 20th epoch with a patience value of 10, which counts sustaining training steps based on the loss for the tuning dataset or the validation loss value, completing training within a total of 100 epochs. During training, if the validation loss value exceeded the minimum validation loss achieved so far in any epoch, the model was not saved. Thus, the model updated at the epoch showing the minimum validation loss in the training process was chosen as the final saved model to prevent overfitting.

The deep-learning model used in this study was implemented on a PyTorch platform using a hardware system comprising an NVIDIA GeForce RTX 4090 graphics processing unit and Intel Xeon Silver central processing unit with a customized water-cooling system.

### Performance evaluation and statistical analysis

After training deep-learning models, the performance of each model was evaluated in the test dataset three times using different seeds. For external validation, the trained deep learning models were tested with another external validation dataset as described above.

The primary outcome measurement for the established deep learning model was the classification accuracy in predicting twelve age groups, delineated on a monthly basis. The secondary outcome included the one-month relaxation accuracy of the deep learning models. Continuous variables are presented as means with standard deviations. Mann–Whitney U test was used for the comparison of prediction performance between different age groups. A P-value of < 0.05 were considered statistically different and all tests were two-sided. A gradient-weighted class activation map (Grad-CAM++) was implemented in the neural network layer to localize the discriminative regions used by the deep-learning tool to determine the specific class in the given images^15^. To validate the superiority of the method proposed in this study, comparison experiments were conducted with the RSNA Bone Challenge Winner Model^16^. RSNA Bone Challenge is a competition for estimating the bone age of pediatric patients based on radiographs of their hand. The RSNA Winner Model used not only InceptionV3 as the deep learning network but also sex as an additional input feature.

### Ethical approval

All procedures performed in studies involving human participants were in accordance with the ethical standards of the institutional and/or national research committee (Institutional Review Board of Hallym University Sacred Hospital and with the 1964 Helsinki declaration and its later amendments or comparable ethical standards.

### Informed consent

This study was carried out as a retrospective analysis, wherein all patient data were anonymized prior to utilization. Informed consent was waived due to the retrospective nature of the study by the Institutional Review Board of Hallym University Sacred Hospital (No. 2023-01-002).

## Results

### Patient characteristics

The entire dataset, including internal and external data, comprised a total of 5797 images from 1552 children. Among those, the dataset included 2401 X-ray images from 1336 patients in the AP view dataset and 2532 images from 1321 patients in the lateral view dataset. The data composition of the training and test datasets is presented in Table 1. The mean age in the internal dataset was 7.3 ± 3.2 months. In the internal dataset, females accounted for 632 (47.1%) of the children, while in the external dataset, 106 individuals (49.1%) were female. The external dataset included 864 images from 216 children.

### Performance of deep learning models for age prediction

**Figure 2:**
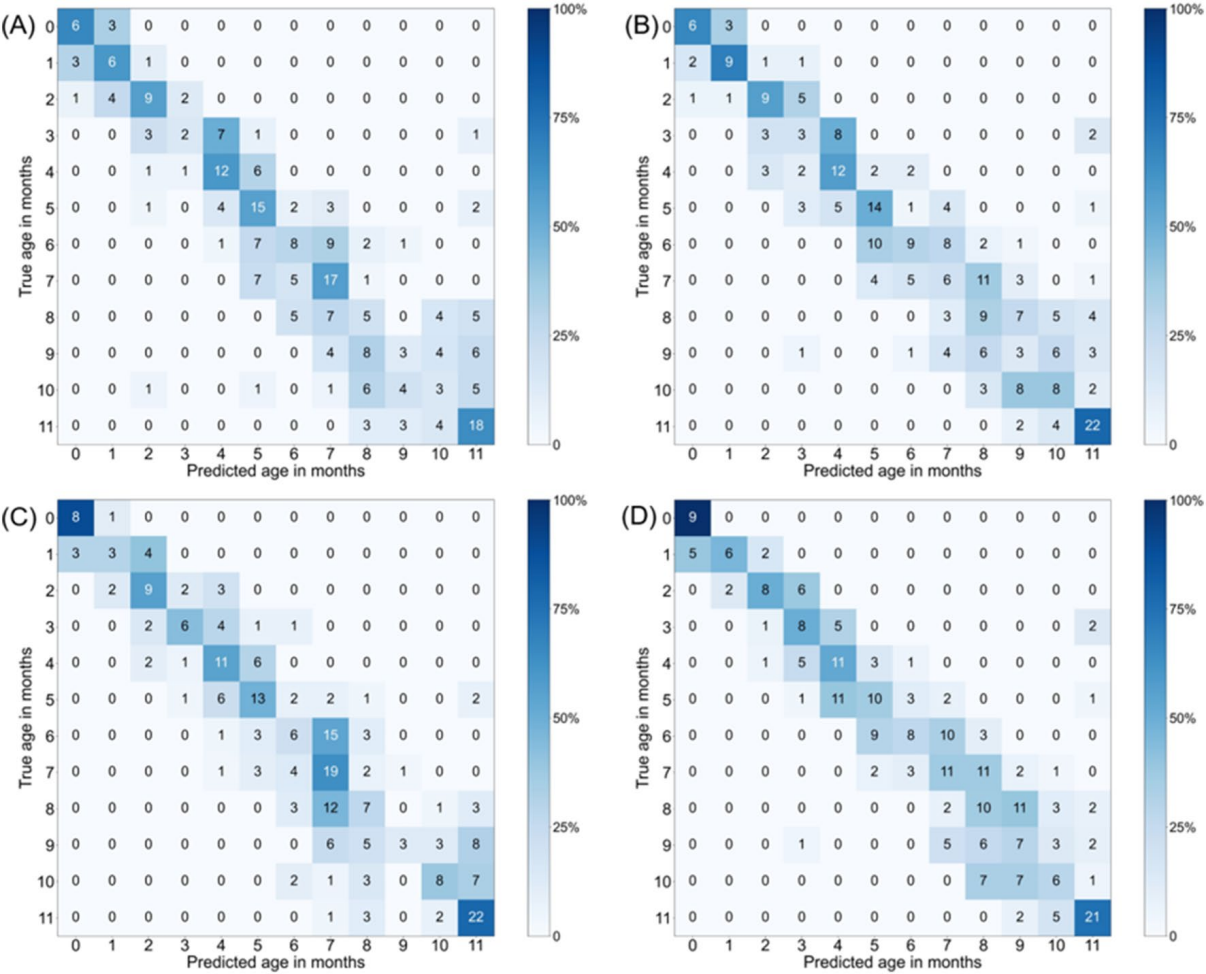
Heatmap of the confusion matrix for the per-month accuracy of DenseNet-121 and EfficientNet-V2-M model for age prediction in the internal test dataset. (**A**) DenseNet-121 for AP view (**B**) DenseNet-121 for lateral view (**C**) EfficientNet-V2-M for AP view (**D**) EfficientNet-V2-M for lateral view.

The prediction performance of deep learning models for the internal and external dataset are presented in Table 2. For the internal dataset evaluation, the accuracy of the DenseNet-121 models in age prediction was 38.5 ± 4.0% for the AP view images and 39.7 ± 1.8% for the lateral view images. The accuracy of the EfficientNet-V2-M models for the internal dataset in age prediction was 39.1 ± 5.5% for the AP view images and 47.8 ± 1.5% for the lateral view images. EfficientNet-V2-M models exhibited 0.6% and 8.1% higher accuracy than DenseNet-121 models for the internal dataset of AP images and lateral images, respectively. The confusion matrices for monthly accuracy are shown in Fig. 2.

**Table 2:** Diagnostic performance for age prediction of machine learning.

View	Model	Internal test set				External test set			
		Accuracy		F1-Score		Accuracy		F1-Score	
		12 age groups	1-month relaxation	12 age groups	1-month relaxation	12 age groups	1-month relaxation	12 age groups	1-month relaxation
AP	DenseNet-121	38.5 ± 4%	78.0 ± 1.5%	0.366 ± 0.147	0.804 ± 0.108	33.9 ± 2.1%	75.5 ± 1.1%	0.332 ± 0.152	0.764 ± 0.148
	EfficientNet-V2-M	39.1 ± 5.5%	77.0 ± 2.3%	0.390 ± 0.159	0.793 ± 0.106	32.8 ± 2.5%	75.3 ± 2.2%	0.298 ± 0.135	0.764 ± 0.158
	InceptionV3 + Sex	31.4 ± 2.1%	66.7 ± 3.4%	0.314 ± 0.023	0.706 ± 0.045	24.9 ± 5.1%	62.4 ± 8.2%	0.226 ± 0.037	0.678 ± 0.071
Lateral	DenseNet-121	39.7 ± 1.8%	81.1 ± 2.9%	0.402 ± 0.142	0.826 ± 0.073	28.3 ± 1.3%	71.1 ± 1.3%	0.268 ± 0.130	0.715 ± 0.148
	EfficientNet-V2-M	43.1 ± 0.5%	85.1 ± 2.5%	0.438 ± 0.150	0.866 ± 0.076	29.5 ± 1.3%	74.1 ± 1.2%	0.304 ± 0.100	0.748 ± 0.132
	InceptionV3 + Sex	32.4 ± 5.6%	73.8 ± 4.2%	0.314 ± 0.088	0.758 ± 0.044	23.4 ± 2.6%	59.9 ± 7.8%	0.215 ± 0.036	0.615 ± 0.060

**Figure 3:**
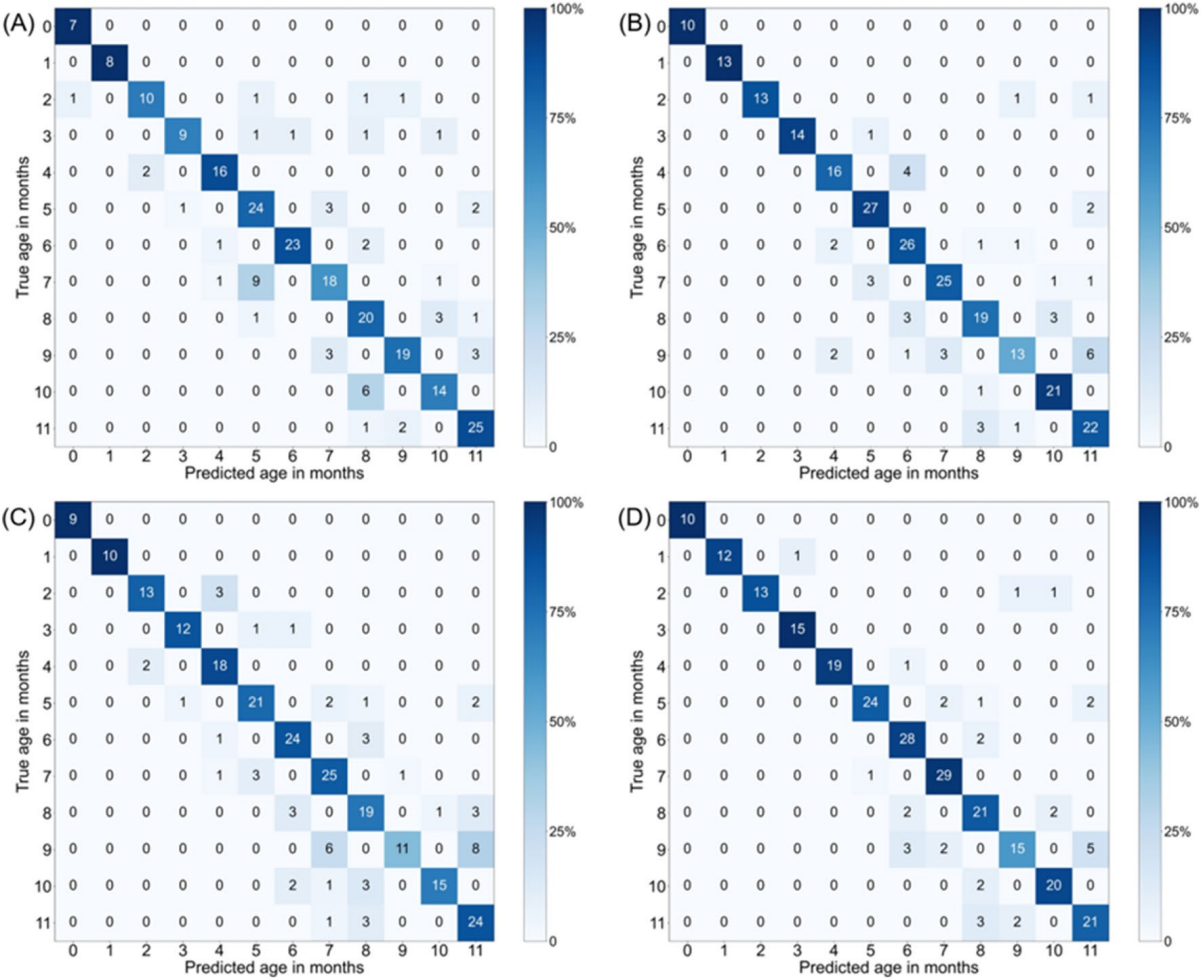
1-month relaxation Results. Heatmap of the confusion matrix for the per-month accuracy of DenseNet-121 and EfficientNet-V2-M model for age prediction in the internal test dataset. (**A**) DenseNet-121 for AP view (**B**) DenseNet-121 for lateral view (**C**) EfficientNet-V2-M for AP view (**D**) EfficientNet-V2-M for lateral view.

When considering a margin of error of ± 1 month, the maximum corrected accuracy of DenseNet-121 for the AP view images reached 78.2%, with an average of 78.0 ± 1.5%, as presented in Fig. 3. For the lateral images, the maximum corrected accuracy under the same error margin was 84.2%, with an average of 81.1 ± 2.9%. On the other hand, for EfficientNet-V2-M, when considering a margin of error of ± 1 month, the maximum corrected accuracy for the AP view images reached 79.1%, with an average of 77.0 ± 2.3%. For the lateral images, the maximum corrected accuracy under the same error margin was 87.3%, with an average of 85.1 ± 2.5%.

To compare the per-class prediction performance by sub-group, the per-class accuracy of EfficientNet-V2-M was analyzed, which performed slightly better overall than DenseNet-121. In terms of 1-month relaxation prediction performance, the accuracy of AP view was highest in the 1-month subgroup (100 ± 0%) and lowest in the 9-month subgroup (51 ± 8%) in the internal dataset, but there was no statistical difference in the per-class prediction performance between two subgroups (p = 0.064). For the lateral view, the accuracy was highest in the 0-month subgroup (100 ± 0%) and lowest in the 9-month subgroup (68 ± 8%), and these values were not statistically different (p = 0.064).

### External validation results

In the evaluation of the external dataset, the accuracy of the DenseNet-121 models in age prediction was 33.9 ± 2.1% for the AP view images and 28.3 ± 1.3% for the lateral view images, as presented in Table 2. The accuracy of the EfficientNet-V2-M models for the external dataset in age prediction was 32.8 ± 2.5% for the AP view images and 29.5 ± 1.3% for the lateral view images. EfficientNet-V2-M models demonstrated 0.7% lower accuracy than DenseNet-121 for the external dataset of AP images and 1.2% higher accuracy than DenseNet-121 models for the external dataset of lateral images. These results indicate that models trained using the internal dataset can predict the ages of skull images in external datasets effectively.

Regarding the one-month relaxation results, for DenseNet-121, when considering a margin of error of ± 1 month, the maximum corrected accuracy for the AP view images reached 76.4%, with an average of 75.5 ± 1.1%. For the lateral images, the maximum corrected accuracy under the same error margin was 72.5%, with an average of 71.1 ± 1.3%. Meanwhile, for EfficientNet-V2-M, when considering a margin of error of ± 1 month, the maximum corrected accuracy for the AP view images reached 77.8%, with an average of 75.3 ± 2.2%. For the lateral images, the maximum corrected accuracy under the same error margin was 75.2% with an average of 74.1 ± 1.2%. There were no statistically significant differences in the F1-scores of 1-month relaxation prediction between DenseNet-121 and EfficientNet-V2-M when using AP view (p = 1.000) and lateral view (P = 0.190) images in the internal dataset, as well as in the external dataset (p = 1.000 and p = 0.081, respectively).

**Figure 4:**
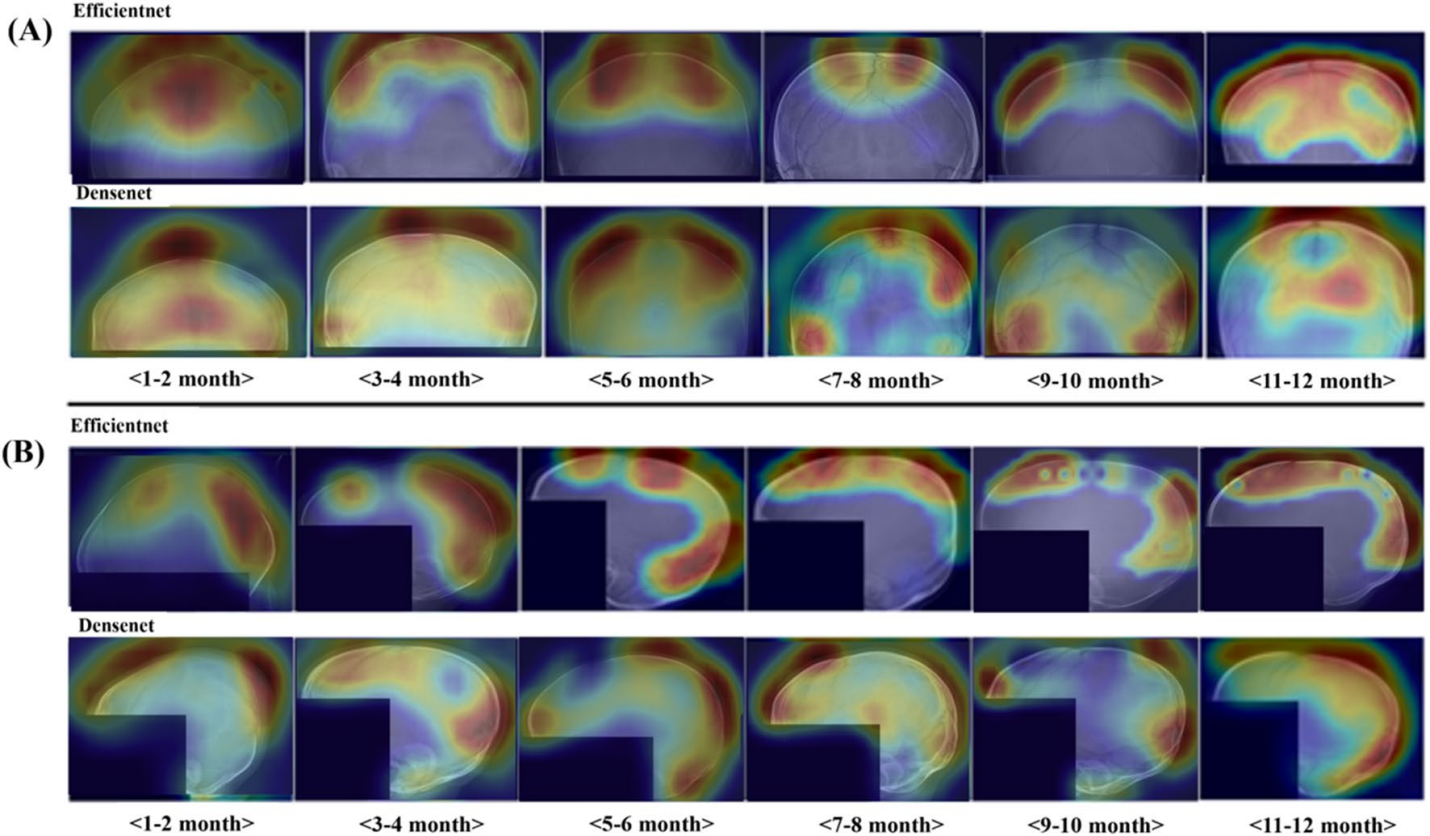
Comparative visualization of majority decision areas at different postnatal stages using Grad-CAM++ in the convolutional neural network algorithm. (**A**, **B**) illustrate radiographic view of the skull: the anteriorposterior and lateral views, respectively. Grad-CAM++ Saliency Maps Highlighting majority decision areas for age classification in Skull X-ray Images. (**A**) Saliency maps for anteroposterior (AP) skull X-ray images across different age categories. The maps show the coronal suture and fontanels as the primary discriminative features used by both EfficientNet and DenseNet models for classifying ages under 12 months. (**B**) Saliency maps for lateral skull X-ray images. These highlight the lambdoid suture and cortical bone density as critical regions for age classification by the models. Each column represents a different age group, providing a visual representation of the features' significance across developmental stages.

To delineate the majority decision areas in classifying age categories, saliency maps were generated using Grad-CAM++. Analysis of these maps revealed that the coronal suture and fontanels in AP skull X-ray images, along with the lambdoid suture and variations in cortical bone density in lateral skull X-ray images, serve as the predominant discriminative regions for age classification within the test dataset (Fig. 4).

### Comparison with the RSNA Bone Challenge winner model

The performances of the RSNA winner model are presented in Table 2. In the prediction performance of 1-month relaxation, the accuracy of the model was 66.7 ± 3.4% for AP view images and 73.8 ± 4.2% for the lateral view images in the internal test dataset. For the external test dataset, the mean accuracy of the RSNA winner model reached 62.4 ± 8.2% for AP view images and 59.9 ± 7.8% for lateral view images. Our best models outperformed the RSNA winner model in the prediction accuracy by approximately 10% on each of the test datasets.

## Discussion

With advances in deep-learning techniques, it has been possible to develop computational models composed of multiple processing layers to learn representations of data with multiple levels of abstraction^17^. To apply deep-learning systems for disease assessment using medical imaging, it is important to realize highly accurate classifications on test datasets as well as reasonable feature extraction of target lesions. However, traditional machine-learning methods for disease classification, such as support vector machines, K-means clustering, and the naïve Bayes classifier, require expert knowledge and time-consuming manual adjustments to extract specific features^18–20^. This implies that traditional machine-learning methods require the extraction of features representing characteristics by using various segmentation methods. Thus, recent deep-learning architectures can facilitate the direct acquisition of useful feature representations from data. The CNN model is known to be a powerful imaging classifier and is widely used to evaluate radiologic images, such as X-rays, computerized tomography, and magnetic resonance imaging^21^. In addition, CAM++ enables classification-trained CNNs to localize characteristic features without using any bounding box annotations^22^. In the present study, using a dataset comprising infantile skull X-ray images, the decision algorithm was shown to be an efficient model for classifying the image data in the age categories.

The observations from GradCAM++ algorithm’s focus provide a valuable tool for estimating an infant's postnatal age by charting the predictable sequence of cranial suture closure and bone development. Furthermore, the descriptive focus of the GradCAM++ algorithm allows for the inference of progressive developmental alterations in skull X-ray images across various postnatal stages (Supplementary Information).

To our knowledge, the present study is the first to develop a deep-learning model for the prediction of infantile age using skull X-rays. The average accuracies achieved by the CNN-based classifier reached 78.0 ± 1.5% for the AP view and 78.0 ± 1.5% for the Lateral view, classifying the age category with a one-month relaxation. Interestingly, we found that decisions pertaining to age classification by CAM++ were based on certain regions in the skull X-ray images: the fontanels and the coronal sutures on the AP images of skull X-rays were used to a remarkable extent while the region of the lambdoid suture and the cortical bone density on the lateral images was also used prominently. We provided explanations that detail the representative morphological hallmarks, as discerned through the GradCAM++ visualizations, that characterize each specified age stage in Table 3. For instance, off-the-midline patterns observed in the Grad-CAM++ Saliency Maps of both the sagittal and metopic sutures are considered indicative of the ongoing ossification of these sutures around the 5–6 postnatal month.

**Table 3:** The representative morphological hallmarks of the descriptive focus from Grad-CAM +  + Saliency Maps.

Post-natal age	Anteriorposterior (AP) view	Lateral view
1–2 months	The visibility of the anterior fontanel and sagittal suture can be correlated with the typical closure times, where the open and broad fontanel indicates a very young age	The wide lambdoid suture and the anterior fontanel gaps are primary indicator of a very young infant as sutures narrow and eventually ossify with age
3–4 months	A shift in attention off the midline, focusing on the sagittal suture, may correspond with the expected morphological changes as the cranial bones begin to grow and the suture starts to ossify	The ascending observation of the lambdoid suture and coronal gap corresponds with the gradual ossification and narrowing of sutures
5–6 months	Further off the midline patterns of the sagittal suture and metopic suture might be indicative of the continuation of ossification and could be used to distinguish between earlier and later stages of infancy	The focus on bone density near the fontanel, rather than the suture patterns, suggests a shift in developmental markers from suture morphology to bone mineralization
7–8 months	Typical observations of the posterior fontanel and lambdoid suture suggest that by this age, the posterior fontanel is beginning to close, a process that usually completes around the second year	Noting the closure of the coronal suture and bone density are indicative of advanced ossification, suggesting an age closer to eight months, as these features do not appear in earlier stages
9–10 months	Revisiting the posterior fontanel and sagittal suture pattern might be indicative of the ongoing ossification process as the infant approaches the end of the first year	Observations extending to bone mineral density above the occipital bone and below the lambdoid suture indicate a progressive state of bone maturation, which is typically expected as the infant approaches one year
11–12 months	The evaluation of the double cortical layer of bone and the visibility of both the coronal and lambdoid sutures suggests a more mature cranial structure, as these features are characteristic of a skull nearing the end of the first postnatal year	The broad examination of the cortical bone and the recognition of the coronal suture closure represent the advanced stage of cranial development usually seen at the end of the first year

Considering the chronological changes during infantile cranial development, CNN-based deep learning effectively identified age categories with reasonable detection of characteristic features representative of cranial development.

The areas highlighted by Grad-CAM++ align with the regions known for characteristic changes in infant skull development over various stages post-birth^23,24^.

Moreover, by examining the Grad-CAM++ areas corresponding to different developmental stages in infants, we were able to deduce retrospectively the specific regions where significant changes occur in the skull development of infants over time. By correlating these observed features with known timelines of cranial development, radiologists and pediatricians can estimate the postnatal age of an infant.

We expect that the novel findings from the present study can be useful in the individual assessment of normal cranial development according to postnatal age. Screening the skull X-ray can be used to detect overdevelopment of the cranial bones compared to the actual postnatal age. Additionally, screening of certain conditions, such as premature closure of the cranial suture, may be possible without computerized tomography of the head, which requires a higher radiation dose. In addition, this method may be helpful in the follow-up of patients who have had surgical correction of craniosynostosis, facilitating assessment of normal cranial development after surgery.

Furthermore, it is expected that the CNN-based deep learning used in the present study can also be applied in legal medicine and archeology. For instance, the developed algorithm could be used in the estimation of the actual age of the corpse at the time of death.

In this study, we presented and evaluated the performance of a deep learning model for age prediction based on cranial development, focusing specifically on cranial sutures and cortical bone development, excluding the facial region of the pediatric skull. However, building on this research, future studies will explore a broader range of cranial metrics, including cephalic index and head circumference, to provide a more nuanced understanding of cranial development. In addition, we plan to explore the integration of these metrics with advanced imaging technologies and machine learning algorithms to improve diagnostic accuracy and prognostic capabilities in cranial pathology.

### Study limitations

The present study is subject to several limitations. Firstly, the deployed deep-learning model was trained exclusively on data from infants aged under 12 month. To enhance the model's applicability, future iterations should incorporate a broader age range, extending to at least 24 postnatal months, with classification conducted on a monthly basis. Secondly, the current deep-learning model was unable to distinguish individual cranial sutures within the skull X-ray images when applying the Grad-CAM++ technique. Subsequent enhancements to this technique that enable precise delineation of each cranial suture will be essential for employing this method in the definitive diagnosis of single-suture craniosynostosis." Thirdly, the variation in spatial resolution of X-rays collected under different circumstances over the past decade could potentially have affected the results of deep learning training. Future research should address these potential effects through a technical evaluation using cranial X-ray data from other institutions over the same time period, using similar equipment and the same X-ray dose. Building upon the algorithm developed in this study, we aim to refine the predictive accuracy concerning infant age, normal cranial development, and pathological cranial anomalies in future research.

### Future perspectives

In the present study, the age information was a numerical variable, and thus the targeted problem could be approached as a regression problem. Nevertheless, we solved it as a classification problem because skull bone development may not simply have features that increase linearly as the continuous variable increases; features may appear in a nonlinear and discontinuous manner, such as sutures disappearing and shapes changing. However, we plan to explore the use of regression in future research.

In the present study, utilizing deep learning, we anticipate the development of algorithms not only for predicting the age of infants under 12 months but also for estimating the age of older children. Additionally, we plan to conduct research aimed at enhancing the accuracy of age prediction by applying the same deep learning algorithm to age-specific CT data of children.

And it will be possible to train a deep learning model using skull X-ray and children's hand bone X-ray or other part of the body's bone X-ray together to predict growth such as height.

## Conclusion

The CNN model developed in the present study showed good performance in predicting the postnatal age categories in the infantile population. Efficient-V2-M model shows better performance than DenseNet-121 model in predicting age of skull for both AP and lateral position images. And we found that deep learning models can predict ages of skulls by viewing cranial sutures that are anatomical meaningful and related to growing pediatrics via analyzing visualizations by Grad-CAM++.

We expect that using plain skull X-rays will help in estimating actual postnatal age and evaluating normal cranial development.

## Supplementary Information

Supplementary Information.

## Data Availability

The authors confirm that the meta-data supporting the results of the deep learning is provided within the supplementary information files.
